# Role of Cholesterol 25-Hydroxylase (Ch25h) in Mediating Innate Immune Responses to *Streptococcus pneumoniae* Infection

**DOI:** 10.3390/cells12040570

**Published:** 2023-02-10

**Authors:** Soo Jung Cho, Alexander Pronko, Jianjun Yang, Kassandra Pagan, Heather Stout-Delgado

**Affiliations:** Weill Cornell Medicine, New York, NY 10021, USA

**Keywords:** *Streptococcus pneumoniae*, Ch25h, alveolar macrophage

## Abstract

Alveolar macrophages (AM) are long-lived tissue-resident innate immune cells of the airways. AM are key effectors of recognition, initiation, and resolution of the host defense against microbes and play an essential role in mediating host responses to *Streptococcus pneumoniae* infection. Lipid metabolism in AM can significantly impact cellular function and biology. Dysregulated metabolism contributes to an accumulation of lipids, unfolded protein response induction, and inflammatory cytokine production. Our study was designed to investigate the impact of Ch25h on mediating innate immune responses by macrophages during *S. pneumoniae infection*. Using wild-type and *Ch25^−/−^* mice, we examined the role of cholesterol metabolism on inflammatory cytokine production and bacterial clearance. Our results demonstrate that Ch25h plays an important role in the initiation and intensity of cytokine and chemokine production in the lung during *S. pneumoniae* infection. In the absence of Ch25h, there was enhanced phagocytosis and bacterial clearance. Taken together, our findings demonstrate the important role of Ch25h in modulating host responsiveness to *S. pneumoniae* infection.

## 1. Introduction

Acute respiratory distress syndrome (ARDS), the most severe form of acute lung injury, is a serious respiratory illness that is commonly caused by bacterial pneumonia, for which a frequently responsible pathogen is *Streptococcus pneumoniae* (*S. pneumoniae*) [1,2]. Alveolar macrophages (AM) are long-lived tissue-resident innate immune cells of the airways. During steady-state conditions, AM adopts a pro-healing, anti-inflammatory phenotype to maintain lung integrity [3]. AM are key effectors of recognition, initiation, and resolution of the host defense against microbes and play an essential role in mediating host responses to *S. pneumoniae* infection [3,4]. Lipid metabolism in AM can significantly impact cellular function and biology. Dysregulated metabolism contributes to an accumulation of lipids, unfolded protein response (UPR) induction, and inflammatory cytokine production [5,6].

A variety of lipids play a critical role in the host’s immune response. Cholesterol 25-hydroxylase (Ch25h) is a redox enzyme, mainly localized in the endoplasmic reticulum (ER) and Golgi apparatus, that catalyzes the oxidation of cholesterol to 25-hydroxycholesterol (25-HC) [7]. 25-HC is an endogenous oxysterol that plays a key role in mediating cholesterol homeostasis and is involved in the regulation of multiple metabolic pathways [8,9]. Previously published work has provided valuable insights into the role of Ch25h as an important regulator of inflammation and immune responses to foreign pathogens. Ch25h expression has been reported in most organs at a steady state to be relatively low. In response to viral infections or TLR-agonist treatment, Ch25h expression is significantly upregulated by *IFNR/JAK/STAT1* signal transduction pathways [10,11,12,13,14,15,16]. Heightened expression of Ch25h and 25-HC has been shown to be associated with increased secretion of cytokines and chemokines [17,18,19]. Several studies have demonstrated that 25-HC can activate NF-kB/NOD2, recruit FOS/JUN, and signal via the ERK1/2 pathway to induce pro-inflammatory mediators [18,20,21,22]. In disease models, such as chronic obstructive pulmonary disease (COPD), Ch25h expression was increased in AM and alveolar cells, with higher levels of Ch25h corresponding to increased IL-8 and neutrophil recruitment in lung tissue [23,24]. During infection, overexpression of Ch25h increased the susceptibility of mice to *Listeria monocytogenes* and *Mycobacterium tuberculosis* and amplified the activation of immune cells and mediators in response to influenza [21,25,26]. Ch25h, by altering membrane cholesterol, has been shown to play a key role in inhibiting viral entry, with Ch25h expression levels being correlated with disease severity [27,28,29]. Further, Ch25h has been shown to promote efferocytosis and resolution of LPS-induced lung injury via LXR-dependent prevention of AM lipid overload [13]. Additional work has demonstrated that interferon signaling can also play a key role in mediating 25-HC production and redistribution of cholesterol [30]. Inhibition of IFN-γ can reprogram cholesterol metabolism and decrease cellular protection against cholesterol-dependent cytolysins (CDC), thereby rendering mice more susceptible to CDC-induced tissue damage [30]. Models of LPS-induced acute lung injury have provided additional insights into a potential dose-dependent role of Ch25h and 25-HC, with increased levels of 25-HC contributing to decreased LPS-induced activation of AM [31]. Based on the current literature, there is a concentration-dependent role for 25-HC and Ch25h to repress or augment the production of host inflammatory responses. Specifically, at low concentrations, 25-HC can dramatically down-regulate pro-inflammatory signaling, with effects decreasing at higher 25-HC concentrations [31]. Despite these insights, very little is currently known about how Ch25h modulates AM responses during *S. pneumoniae* infection. Our study was designed to investigate the impact of Ch25h on mediating innate immune responses by macrophages during infection by *S. pneumoniae* infection. Our results demonstrate that Ch25h plays an important role in the initiation and intensity of cytokine and chemokine production in the lung during *S. pneumoniae* infection. Knockdown of *Ch25h* gene expression resulted in enhanced phagocytosis and bacterial clearance. Taken together, our findings demonstrate the important role of Ch25h in modulating host immune responses to *S. pneumoniae* infection.

## 2. Materials and Methods

### 2.1. Mice

Male and female wild-type (C57BL/6, Jackson Labs: strain #: 000664) and *Ch25h^−/−^* (Jackson Labs: strain # 016263) were bred in the WCM animal facility and mice were handled under identical husbandry conditions and fed certified commercial feed. To control for microbiome differences, mice were co-housed in the same room and rack of the facility. The IACUC at Weill Cornell Medicine approved the use of animals in this study and methods were carried out in accordance with the relevant guidelines and regulations. Sex- and age-matched littermates were used for the proposed experiments. Mice were used at 3 months of age and no animals were used in the study if there was evidence of skin lesions, weight loss, or lymphadenopathy.

### 2.2. Streptococcus pneumoniae Infection

For in vitro and in vivo experiments, serotype 3 strain, ATCC 6303 (American Type Culture Collection) was used. Briefly, prior to infection, ATCC 6303 was grown on 10% sheep blood agar plates (BD Biosciences) overnight. Colonies were isolated and grown in Todd Hewitt Broth (THB) containing 2% yeast extract until OD600 was 0.5. *Bacterial binding assay*: Bacterial binding was performed on cells freshly washed with PBS. Cells were collected and centrifuged for 10 min at 500× *g* prior to incubation with *S. pneumoniae* (MOI = 10). Cells were incubated for 1 h (4 °C) to allow for binding. Cells were incubated for an additional 60 min. Bacteria bound to cells were pelleted at 500× *g* for 5 min (4 °C). Cells were washed twice with PBS prior to macrophage resuspension (1 mL of PBS) and lysis (100 μL of macrophage solution in 900 μL of sterile water). Samples were serially diluted to determine the CFU. *Gentamicin protection assay*: Cells were collected and centrifuged prior to incubation with *S. pneumoniae* (MOI = 10). Cells were incubated for 30 min at 37 °C to allow for bacterial binding. Cells were incubated for an additional 60 min, prior to centrifugation (500× *g*, 5 min), removal of supernatant, and subsequent addition of 1X HBSS containing 50 μg/mL gentamicin. Cells were incubated at 37 °C for 40 min prior to centrifugation and washed with fresh 1X HBSS. Macrophages were resuspended in 1 mL of PBS prior to lysis (100 μL of macrophage suspension added to 900 μL of sterile water). Samples were serially diluted to determine the CFU.

### 2.3. Cell Isolation and Culture

Alveolar macrophages (AM) were isolated from freshly isolated lung tissue. Briefly, lung tissue was dissociated per manufacturer’s instructions using the Lung Dissociation kit (Miltenyi Biotec, Bergisch Gladbach, Germany). CD11b^+^ cells were depleted using CD11b MicroBeads followed by depletion using LD columns (Miltenyi Biotec). Using the CD11b depleted fraction, CD64^+^ cells were enriched following incubation with anti-mouse PE-conjugated REAfinity CD64 antibody and secondary conjugation with anti-PE microbeads (Miltenyi Biotec). Cells were collected using LS columns. To confirm purity of the macrophage population, the percentage of Siglec F^+^ CD64^+^ CD11c^+^ CD11b^−^ cells were assessed by flow cytometry using previously published gating strategies and found to be >95% of the isolated population [32]. AM populations were allowed to rest for four hours prior to each experiment.

### 2.4. Bacterial Labeling

*Alexa Fluor 647/FITC labeling*: *S. pneumoniae* was grown at 37 °C (5% CO_2_) for 4 h (0.5, OD 600) prior to pelleting by centrifugation (15,000× *g*, 1 min). Bacteria were washed once in sterile PBS. Diluted FITC-ester (10 mg/mL) and Alexa Fluo 647 (1 mg/mL) (ThermoFisher Scientific) was added to 3 × 10^7–8^ bacteria/mL of PBS. Suspensions were incubated in the dark for one hour at room temperature with continuous vortexing (medium speed). Labeled *S. pneumoniae* was washed 3× in PBS and resuspended to desired concentration. Macrophages were treated with MOI = 10 labeled *S. pneumoniae* (diluted in PBS) and incubated at 4 °C to allow for bacterial binding between all samples. Samples were centrifuged and washed with 1× PBS prior to incubation at 37 °C for 30-, 60-, or 120-min. Macrophages were centrifuged at 500× *g* for 5 min prior to washing with PBS and resuspension in 250 mL of 1× PBS. Fluorescence was quantified using the BD Accuri c6 cytometer and analyzed by FlowJo Software. Representative gating strategy for analysis is shown in Supplemental Figure S1.

### 2.5. In Vivo Procedures and Tissue Collection

*Streptococcus pneumoniae infection*: *Streptococcus pneumoniae* was plated one day prior to growth on tryptic soy agar plates containing 5% sheep blood (Becton Dickenson, Franklin Lakes, NJ, USA). Colonies were isolated and grown for 3 h in Todd Hewitt Broth (THB) containing 2% yeast extract (37 °C, 5% CO_2_). All mice were anesthetized with isoflurane (5% for induction and 2% for maintenance) prior to intranasal instillation with PBS or 1 × 10^5^ CFU of *S. pneumoniae* (50-μL volume in PBS). Body weights were measured daily, and mice were humanely euthanized if they lost more than 15% of their starting body weight. *Bronchoalveolar lavage (BAL):* BAL was collected using previously published methods [33]. Briefly, 0.8 mL of PBS was slowly injected and aspirated 4 times prior to saving the recovered lavage fluid on ice. Lavage was clarified at 1200 rpm for 10 min at 4 °C. *Protein quantification in BAL*: Protein levels in clarified lavage were calculated using the BioRad protein assay (BioRad, Contra Costa County, CA, USA) per manufacturer’s instructions. *Bacterial titer assay of lung tissue*: Lung tissue samples were collected at select time points of infection and digested prior to serial dilution in THB. Samples were serially diluted and plated on blood agar plates prior to overnight incubation at 37 °C, 5% CO_2_. Colony formation was quantified and CFU was normalized to lung weight (grams). *FITC-Dextran Lung Permeability Assay*: Young and aged adult mice were intranasally instilled with 50 μL of FITC-Dextran (3 mg/kg). After 1 h, blood was collected from euthanized mice, and plasma was isolated after centrifugation (7000 rpm, 10 min). Fluorescence was assessed (excitation 485, emission 528). *Histology:* Mice were euthanized, and right lung tissue was collected for downstream analysis. To maintain architecture, left lung was distended with 1% low melting agarose and placed into cold formalin [34]. Tissue samples were processed, and H&E stained by the Translational Research Program at WCM Pathology and Laboratory of Medicine. Images were scanned using the EVOS FL Auto Imaging System (Thermo Fisher Scientific, Waltham, MA, USA). For all animal experiments, we used 5–10 mice per group, and experiments were repeated at least three times.

### 2.6. RNA Purification and Real-Time PCR

RNA samples were extracted using the automated Maxwell RNA extraction protocol (Madison, WI, USA). Samples were quantified and A_260/280_ ratios were recorded. Samples were reverse transcribed using the First Stand Synthesis Kit and quantified RT^2^ Profiler^TM^ Assays (Qiagen: Mouse Antibacterial Response, PAMM-148Z, and Mouse Phagocytosis Array, PAMM-173Z). PCR efficiency, reproducibility, and fold change in expression were quantified using online analytical software provided by Qiagen Gene Globe.

### 2.7. Cytokine Quantification in Lung Tissue

Lung tissue samples were analyzed semi-quantitatively using the mouse XL Cytokine Array Kit (R&D Systems) to assess inflammatory mediator levels at baseline, 24, and 72 h post-infection. Samples were incubated with spotted nitrocellulose membranes according to the manufacturer’s instructions. Protein concentration of samples was determined by BCA assay prior to loading samples and normalized total protein content (~200 µg total protein). Membrane HPR luminescence intensities were quantified using the Quick Spots software (Western Vision). Negative control and reference spots were quantified in each experiment to validate the analysis. Three biological replicates were measured per condition.

### 2.8. Transmission Electron Microscopy (TEM)

The fixative for TEM sample preparation was composed of 4% paraformaldehyde, 2.5% glutaraldehyde, 0.02% picric acid in 0.1M sodium cacodylate buffer (pH 7.3). Cells were washed once in PBS prior to addition of 2 mL of TEM fixative prior to submission to the WCM Microscopy and Image Analysis Core Facility for sample processing and image acquisition. A Jeol electron microscope (JEM-1400) was used to obtain images with accelerating voltage of 100 kV.

### 2.9. Statistical Analysis

Survival analysis between groups was calculated using the Mantel–Cox test. Comparison of groups was performed using a two-tailed t-test and comparisons between groups were verified by one-way ANOVA. For two component comparisons (time post-infection and age), two-way ANOVA was used to calculate statistical significance. All samples were independent and contained the same sample size for analysis. All data were analyzed using GraphPad Prism software (San Diego, CA, USA). Statistical significance was considered by a * *p* < 0.05, ** *p* < 0.01, *** *p* < 0.001, and **** *p* < 0.0001.

## 3. Results

Given the importance of Ch25h in modulating inflammation, the overarching goal of our current study was to examine the role of Ch25h in modulating host responses to *S. pneumoniae* infection.

### 3.1. Decreased Lung Injury in Ch25h^−/−^ Mice during S. pneumoniae Infection

**Figure 1:**
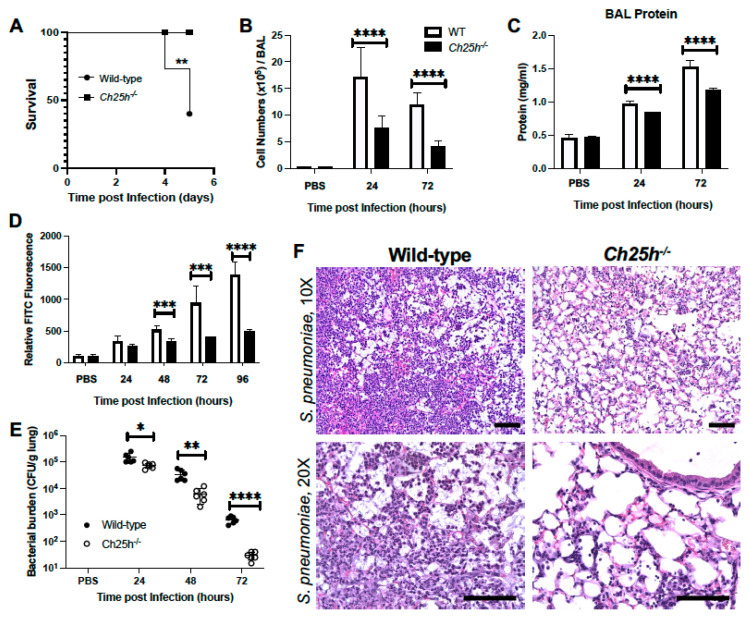
Decreased lung injury in *Ch25h^−/−^* mice during *S. pneumoniae* infection. Wild-type and *Ch25h^−/−^* mice (3 months of age) were intranasally instilled with PBS or 1 × 10^5^ CFU of *S. pneumoniae*. All mice receiving PBS survived. (**A**) Survival was assessed in both groups. BAL was collected and (**B**) total cell number and (**C**) protein levels were quantified. (**D**) At select time points post-infection, mice were instilled with FITC (3 mg/kg in 50 mL) prior to isolation of plasma. Fluorescence in plasma was assessed. (**E**) CFU was assessed in serially diluted lung tissue collected at select time points of infection. (**F**) Lung tissue was collected at 72 h post instillation. Representative photomicrographs (hematoxylin and eosin stained) taken from each group were shown (magnification of ×10 and ×20). Scale bars: 20 μm. N *=* 5–10 per group, * *p* < 0.05, ** *p* < 0.01, *** *p* < 0.001, **** *p* < 0.0001. Results were repeated at least twice with representative results shown.

Wild-type and *Ch25h^−/−^* mice were instilled with PBS or *S. pneumoniae* (1 × 10^5^ CFU) on day 0. There was a significant increase in survival, as *Ch25h^−/−^* were protected from lethality during infection (Figure 1A). We next examined cellular infiltration and observed in the absence of Ch25h, there were decreased BAL cellular numbers (Figure 1B). To investigate the impact of Ch25h on lung permeability, we quantified protein levels in BAL as well as FITC migration from lung to plasma. Ch25h knockdown corresponded with decreased BAL protein and FITC fluorescence in plasma (Figure 1C,D). We next examined the impact of *Ch25h^−/−^* on bacterial clearance. In the absence of Ch25h, we observed a significant increase in clearance of *S. pneumoniae* from the lung at 24, 48, and 72 h post-infection (Figure 1E). Histological examination of lung tissue demonstrated a notable increase in inflammation and intra-alveolar edema detected in wild-type lungs at 72 h of infection (Figure 1F). In the absence of Ch25h, there was decreased cellular recruitment to the lung, with detectable damage to the alveolar–capillary barrier (Figure 1F).

### 3.2. Decreased Chemokine and Inflammatory Cytokine Expression in Ch25h^−/−^ Lung during S. pneumoniae Infection

**Figure 2:**
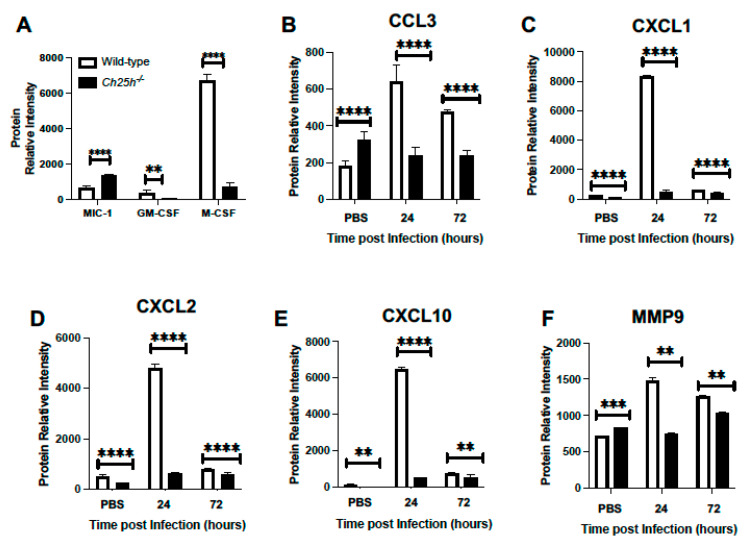
Decreased chemokine expression in *Ch25h^−/−^* lung during *S. pneumoniae* infection. Wild-type and *Ch25h^−/−^* mice were intranasally instilled with PBS or 1 × 10^5^ CFU of *S. pneumoniae*. Lung tissue was collected from PBS instilled (24 h) and in response to *S. pneumoniae* (24 and 72 h). Production of (**A**) MIC-1/GDF-15, GM-CSF, and M-CSF, (**B**) CCL3 (**C**) CXCL1, (**D**) CXCL2, (**E**) CXCL10, and (**F**) MMP9 was assessed in lung homogenates by cytokine array. N = 3 samples per group run in duplicate, ** *p* < 0.01, *** *p* < 0.001, **** *p* < 0.0001.

We next examined the role of Ch25h on chemokine expression in the lung during *S. pneumoniae* infection. Lung tissue was collected from PBS controls and *S. pneumoniae*-infected lungs (24 and 72 h post-instillation). At 24 h post-infection, we observed a significant increase in macrophage inhibitory cytokine 1 (GDF-15/MIC-1) in the absence of Ch25h (Figure 2A). In contrast, there was decreased granulocyte-macrophage colony-stimulating factor (GM-CSF) and macrophage colony-stimulating factor (M-CSF) in *Ch25h^−/−^* lung at 24 h post-infection (Figure 2A). We next quantified the expression of additional chemokine ligands in wild-type and *Ch25h^−/−^* lung at baseline and in response to *S. pneumoniae* infection. We observed a Ch25h dependent increase in CCL3, CXCL1, CXCL2, and CXCL10, which had a similar expression pattern with heightened MMP9 expression (Figure 2B–F).

**Figure 3:**
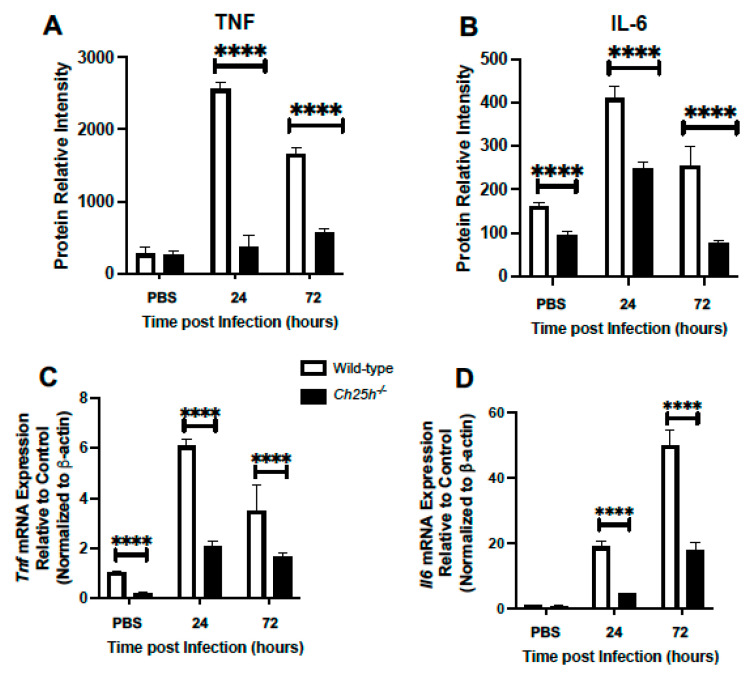
Decreased TNF and IL-6 expression in *Ch25h^−/−^* lung and macrophages during *S. pneumoniae* infection. (**A**–**D**) Wild-type and *Ch25h^−/−^* mice were intranasally instilled with PBS or 1 × 10^5^ CFU of *S. pneumoniae*. (**A**,**B**) Lung tissue was collected from PBS instilled (24 h) and in response to *S. pneumoniae* (24 and 72 h). Production of (**A**) TNF and (**B**) IL-6 was assessed in lung homogenates by cytokine array. N = 3 samples per group run in duplicate. (**C**,**D**) alveolar macrophages were collected from PBS instilled (24 h) and in response to *S. pneumoniae* (24 and 72 h). (**C**) *Tnf* and (**D**) *Il6* gene expression was assessed. For cytokine array experiments, N = 3, repeated in duplicate. For alveolar macrophage experiments, N = 5 per group with results being repeated at least three times. **** *p* < 0.0001.

Given these findings, we evaluated if decreased cellular infiltration and chemokine production might be associated with changes in TNF or IL-6 production. In response to *S. pneumoniae* infection, a significant reduction of TNF and IL-6 was observed in the absence of Ch25h (Figure 3A,B). We next examined the impact of *Ch25h*^−/−^ on *Tnf* and *Il6* gene expression in AM isolated from the lung at select time points of *S. pneumoniae* infection. There was significantly diminished *Tnf* and *Il6* expression observed in AM collected from *Ch25h^−/−^* lung (Figure 3C,D).

### 3.3. Decreased IL-1β Production and Inflammasome Gene Expression in Ch25h^−/−^ Macrophages

**Figure 4:**
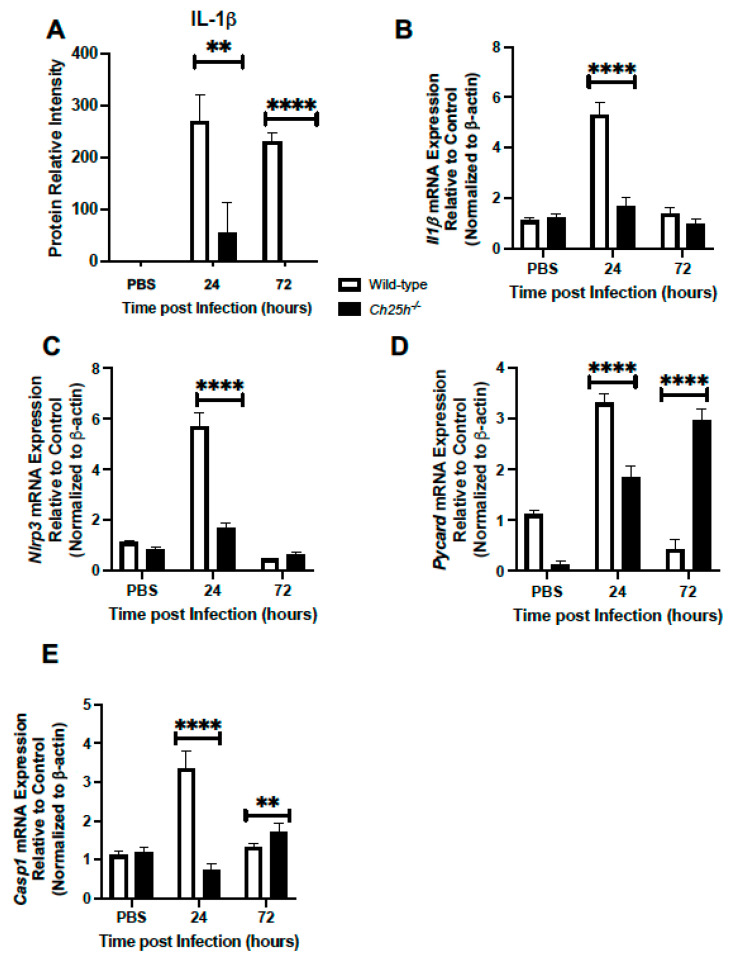
Decreased IL-1β production and inflammasome gene expression in *Ch25h^−/−^* macrophages. (**A**) Wild-type and *Ch25h^−/−^* mice were intranasally instilled with PBS or 1 × 10^5^ CFU of *S. pneumoniae*. Lung tissue was collected from PBS controls (24 h) and in response to *S. pneumoniae* (24 and 72 h). Protein was isolated from lung and IL-1β expression was assessed by cytokine array. (**B**–**D**) Alveolar macrophages were isolated from infected lung at select time of *S. pneumoniae* infection. mRNA was extracted and *Il1β*, *Nlrp3*, *Pycard*, and *Casp1* gene expression was assessed by real-time PCR. For cytokine array experiments, N = 3, repeated in duplicate. For alveolar macrophage experiments, N = 5 per group with results being repeated at least three times. ** *p* < 0.01 and **** *p <* 0.0001.

Given the importance of the inflammasome in modulating host responses to *S. pneumoniae*, we next evaluated if decreased *Ch25h* expression might impact IL-1β secretion and expression of inflammasome components, *Nlrp3*, *Pycard*, and *Casp1*. In response to *S. pneumoniae*, we observed diminished IL-1β production in *Ch25h^−/−^* lung tissue isolated at 24 and 72 h post-infection (Figure 4A). We next evaluated the potential impact of *Ch25h* on *Il1β* expression and observed significantly lower levels in *Ch25h^−/−^* alveolar macrophages (Figure 4B). To determine the impact of Ch25h on AM-specific NLRP3 inflammasome signaling, we isolated AMs from PBS and *S. pneumoniae* instilled lung and assessed gene expression at select time points of infection. In response to infection, there was a significant, Ch25h-dependent increase in *Nlrp3, Pycard*, and *Casp1* gene expression observed at 24 h post-infection (Figure 4C–E). Interestingly, by 72 h of infection, in the absence of Ch25h, there was a significant increase in *Pycard* and *Casp1* gene expression observed (Figure 4D,E).

### 3.4. Enhanced Phagocytosis Receptor Expression by Ch25h^−/−^ Macrophages during S. pneumoniae Infection

**Figure 5:**
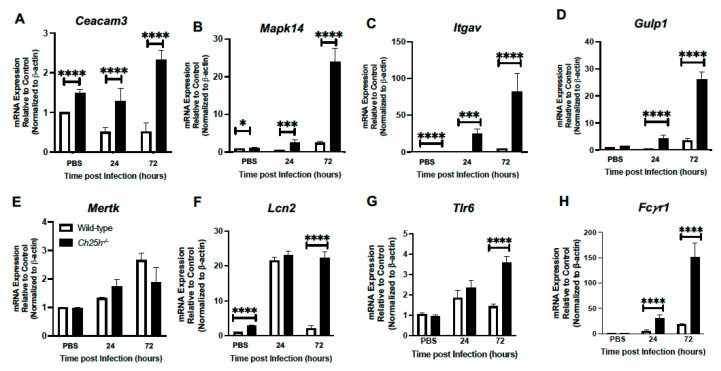
Increased phagocytosis receptor expression in *Ch25h^−/−^* alveolar macrophages isolated during *S. pneumoniae* infection. Wild-type and *Ch25h^−/−^* mice were intranasally instilled with PBS or 1 × 10^5^ CFU of *S. pneumoniae*. Alveolar macrophages were isolated from PBS controls (24 h) and in response to *S. pneumoniae* (24 and 72 h). mRNA was isolated and (**A**) *Ceacam3*, (**B**) *Mapk14*, (**C**) *Itgav*, (**D**) *Gulp1*, (**E**) *Mertk*, (**F**) *Lcn2*, (**G**) *Tlr6*, and (**H**) *Fcrγ1* gene expression was assessed by real-time PCR. N = 5 per group, * *p* < 0.1, *** *p* < 0.001 and **** *p* < 0.0001. Results were repeated four times with representative results shown.

We next examined if phagocytosis by macrophages in response to *S. pneumoniae* might be altered in the absence of Ch25h. To this extent, we isolated alveolar macrophages from wild-type and *Ch25h^−/−^* lung in response to PBS (24 h) or at 24 and 72 h post-*S. pneumoniae* infection. In the absence of Ch25h, there was a significant increase in *Ceacam3* (Figure 5A), *Mapk14* (Figure 5B), *Itgav*, (Figure 5C), and *Gulp1* (Figure 5D) in response to *S. pneumoniae* infection. Despite these findings, we did not observe a significant difference in *Mertk* expression in these AM (Figure 5E). In response to *S. pneumoniae*, there was a similar expression of *Lcn2* (Figure 5F) and *Tlr6* (Figure 5G) quantified at 24 h, with significantly elevated levels present in *Ch25h^−/−^* alveolar macrophages at 72 h. As AMs can bind targets opsonized with immunoglobulin through Fcγ receptors, we next examined the impact of Ch25h on *Fcγr1* expression. In response to *S. pneumoniae* infection, there was an increase in *Fcγr1* levels in wild-type and *Ch25h^−/−^* AM, with significantly elevated levels present in the absence of Ch25h (Figure 5H).

**Figure 6:**
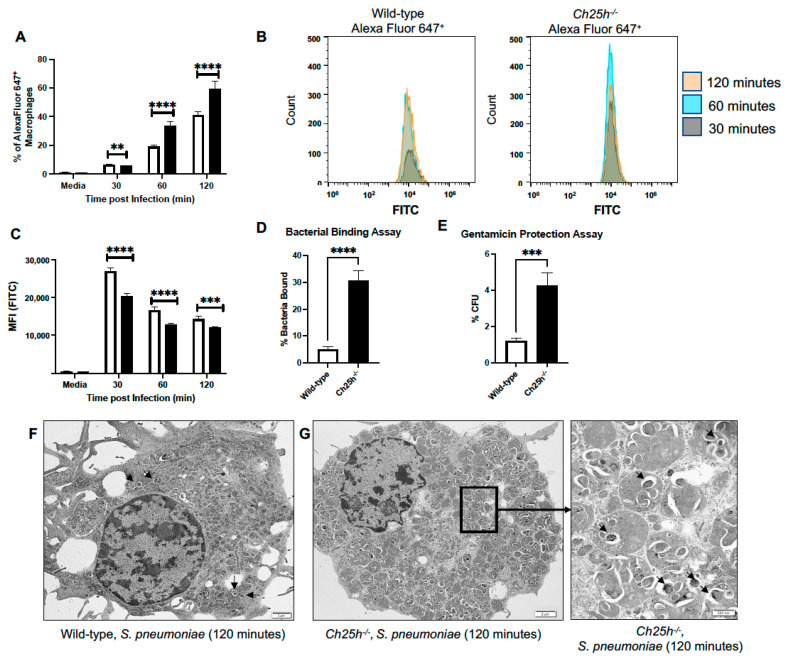
Increased *S. pneumoniae* phagocytosis and clearance by *Ch25h^−/−^* macrophages. Alveolar macrophages were isolated from untreated/uninfected lung. After 4 h, macrophages were infected for select time points with (**A**–**C**) FITC/Alexa 647^+^ labeled *S. pneumoniae* (MOI = 10) prior to analysis by flow cytometry. (**A**) % of Alexa Fluor 647^+^ macrophages were quantified. (**B**,**C**) FITC fluorescence was assessed in Alexa Fluor 647^+^ macrophage populations. (**D**) Bacterial binding and (**E**) gentamicin protection assays were performed using freshly isolated alveolar macrophages. Representative TEM images of *S. pneumoniae* infected (**F**) wild-type and (**G**) *Ch25h^−/−^* alveolar macrophages (120 min). (**F**,**G**) Examples of bacteria are marked by arrowheads. Bar = 2 mm, insert (**G**) Bar = 500 nm. N = 5 per group, ** *p* < 0.01, *** *p* < 0.001 and **** *p* < 0.0001. Results were repeated at least three times with representative results shown.

We examined the role of Ch25h on phagocytosis and *S. pneumoniae* clearance. Wild-type and *Ch25h^−/−^* AMs were cultured with FITC/Alexa Fluor 647^+^ labeled *S. pneumoniae*. At select time points, cells were washed, and phagocytic uptake (Alexa Fluor 647^+^ cells) and processing (FITC fluorescence) was measured by flow cytometry (Figure 6A,B). In the absence of Ch25h, there was a significant increase in the percentage of Alexa Fluor 647^+^ macrophages observed at 60 and 120 min (Figure 6A). Within the Alexa Fluor 647^+^ macrophages, changes in FITC fluorescence were quantified. In the absence of Ch25h there was a significant change in FITC fluorescence intensity observed (Figure 6B,C). To investigate if changes in *S. pneumoniae* processing might be due to Ch25h mediated alterations in bacterial binding and uptake, we performed bacterial binding and gentamicin protection assays using primary wild-type and *Ch25h^−/−^* AM. There was a significant increase in bacterial binding (Figure 6D) and *S. pneumoniae* uptake (Figure 6E) that occurred in the absence of Ch25h. To further these findings, we treated wild-type and *Ch25h^−/−^* alveolar macrophages with *S. pneumoniae* and examined uptake using transmission electron microscopy. We did not observe any phenotypic differences between wild-type and *Ch25h^−/−^* macrophages treated with media (Supplemental Figure S2). In agreement with our flow cytometry findings, in response to *S. pneumoniae* infection, there was an increase in bacteria observed in *Ch25h^−/−^* macrophages when compared to wild-type (Figure 6F,G).

## 4. Discussion

Ch25h has been previously shown to be an important regulator of inflammation and immune responses. The results of our current study expand upon these findings and demonstrate that in addition to other pathogens, Ch25h plays a critical role in modulating host immune responses to *S. pneumoniae* infection.

Previous work has demonstrated that Ch25h-deficient macrophages overproduce IL-1β and have deregulated caspase-1-activating inflammasome activity in response to *Listeria monocytogenes* infection or LPS stimulation [35]. Upregulation of Ch25h during *L. monocytogenes* infection and in response to type I IFN treatment can lead to the repression of caspase 1 activation [26]. The suppressive effect of 25-HC has been shown to be dependent on the repression of SREBP processing and it is plausible that altered cellular lipid content downstream of SREBP activity might contribute to changes in inflammasome activation [35]. Importantly, overexpression of Ch25h has been shown to increase the susceptibility of mice to *L. monocytogenes* and *Mycobacterium tuberculosis* and can amplify the activation of immune cells and inflammatory mediators [21,25,26]. Given the unique ability of *L. monocytogenes* to escape endosomes and survive in host cytosol, it is possible that it has evolved specific adaptations to deal with cytosolic stress and to combat Ch25h mediated induction of host responses. The results of our current study demonstrate that in the absence of Ch25h, there was a significant decrease in IL-1β expression during *S. pneumoniae* infection that was associated with decreased *Nlrp3*, *Pycard,* and *Casp1* expression. It is plausible that Ch25h participates in a transcriptional positive feed-forward loop that amplifies stimulation of the NLRP3 inflammasome during *S. pneumoniae* infection. Previous work has demonstrated decreased responsiveness of *Ch25h^−/−^* macrophages to MYD88-dependent activation in response to TLR7 or 9 stimulations [21]. In this context, Ch25h may attenuate the transcriptional response to *S. pneumoniae* infection.

Previous work has shown that 25-HC can augment macrophage and epithelial cell secretion of IL-6 and M-CSF [21]. In agreement with these studies, in the absence of Ch25h and thereby a reduction in 25-HC, we observed decreased M-CSF and significantly diminished levels of TNF and IL-6 production in the lung during *S. pneumoniae* infection. Based on our current findings and experimental observations, it might be possible that in the absence of Ch25h, 25-HC mediated transcriptional responses, and subsequent inflammatory signaling was dampened. These findings would agree with those observed in studies examining the potential role of Ch25h in mediating host responses to influenza [21]. Examination of the contribution of Ch25h to macrophage-specific production of TNF and IL-6 demonstrated that in the absence of Ch25h, there were decreased *Tnf* and *Il6* transcripts present in alveolar and bone marrow-derived macrophages during *S. pneumoniae* infection. Although the mechanisms by which 25-HC modulates IL-6 and M-CSF have not been fully elucidated, it has been postulated that changes in *NF-kB* signaling due to increased AP-1 promotor binding might contribute to these inflammatory responses [21]. It is also possible that diminished inflammatory responses by Ch25h deficient macrophages may contribute to decreased neutrophil recruitment and neutrophil elastase-mediated injury to the lung. A recent study has illustrated that while pneumolysin exerted minimal cytotoxicity against macrophages, the release of neutrophil elastase by neutrophils impaired phagocytic activity in macrophages [36]. Thereby, decreased Ch25h mediated inflammatory signaling and neutrophil recruitment to the lung would result in less neutrophil elastase-mediated impairment of macrophage phagocytosis. Future work will need to be performed to fully characterize the role by which 25-HC modulates these responses.

Bacteria and viruses often alter cellular lipid metabolism to support their replication. Recent work has illustrated that infection by bacterial species, such as *L. monocytogenes* and *Shigella flexneri*, is suppressed by 25-HC mediated mobilization of accessible cholesterol from the plasma membrane [37]. Importantly, 25-HC has been shown to suppress contact-dependent epithelial cell-to-cell pathogen spread [37]. Elimination of accessible cholesterol by 25-HC provided protection against intercellular dissemination of *L. monocytogenes* and *S. flexneri* [37]. Thereby, the ability of 25-HC to regulate the synthesis and compartmentalization of lipids may enable it to have additional antibacterial effects. In the absence of Ch25h, an increase in intracellular cholesterol levels due to decreased 25-HC production resulted in elevated phagocytosis. This may be attributed to increased lipid packing and changes in membrane permeability, which in turn may contribute to increased pathogen uptake. While our study focused on macrophage-specific responses in the absence of Ch25h, it is possible that changes in 25-HC mediated cholesterol metabolism in alveolar epithelial cells might significantly modulate *S. pneumoniae* permissibility in the lung. Future studies will need to be performed to fully disentangle the role of Ch25h and 25-HC on epithelial cell cholesterol metabolism and host susceptibility to *S. pneumoniae* infection.

It is important to note that experimental findings in the current study demonstrate decreased bacterial burden in lung tissue collected from *Ch25h^−/−^* mice. Previous work has illustrated variations within the *S. pneumoniae* infection model, with differences in lethality and bacterial burden observed using strain ATCC 6303 [38,39,40,41]. In comparison with previous studies, we observed a similar trend in the survival of wild-type mice within the first 5 days of infection [40]. However, we did not observe the same lethality curve as demonstrated in other studies [38,41]. One possibility for these differences might be attributed to the length of *S. pneumoniae* culture prior to instillation. Previous work has demonstrated that pneumolysin release by serotype 3 *S. pneumoniae* strongly increased when reaching the end of logarithmic growth (around 6 h) [42]. Significantly higher levels of extracellular pneumolysin can be readily detected in culture and may be present when the inoculum was prepared for instillation [42]. Studies that recorded increased lethality during infection used bacteria that had been cultured for 6–8 h prior to instillation, while studies with reduced lethality used bacteria cultured for 2 h [38,40,41]. For our current study, we chose to use 3 h of bacterial culture prior to instillation and observed a 5-day lethality curve similar to previous studies with shorter bacterial culture incubations prior to instillation [40].

Previous work has demonstrated that neutrophil complement receptor, CR3, was the primary trigger for phagocytosis of iC3b ligands present on serotypes 6A and 14 bearing pneumococci [43]. Despite serotype 3 strains of *S. pneumoniae* bearing C3b, C3d, and iC3b ligands on the capsule, CR3-mediated phagocytosis by neutrophils only accounted for 20% of bacterial uptake [43]. Additional studies have illustrated that pre-treatment of ATCC 6303 with trypsin can reduce bacterial virulence upon intraperitoneal challenge [44]. Importantly, trypsin did not have any deleterious effect on the polysaccharide capsule and pre-treatment further supported that surface proteins play an important role in phagocytosis resistance by neutrophils [44]. Taken together, these findings demonstrate that serotype 3 *S. pneumoniae* can strongly resist phagocytosis by neutrophils. In our current study, we observe phagocytosis of *S. pneumoniae* by alveolar macrophages to occur within 120 min of culture. This may be attributed to the direct recognition of PAMPs at the macrophage surface or binding of targets opsonized with immunoglobulin through Fcg receptors. Results of our current study demonstrate elevated levels of phagocytosis receptors, as well as Fcg receptor expression, occurred in both wild-type and *Ch25h^−/−^* alveolar macrophages, with higher expression observed in the absence of Ch25h. Given the unique contribution of each cell type to innate immune responses, it may be possible that these findings reflect the diverse sensitivity of different immune cell populations in the lung to foreign pathogens.

Additional work has demonstrated that newly synthesized cholesterol, rather than total cholesterol available in the plasma membrane, was linked to the sensitivity or resistance of macrophages to cholesterol-dependent cytolysin (CDC)-mediated toxicity [30]. Specifically, resistance to CDC-induced pore formation required the production of 25-HC, inhibition of cholesterol synthesis, and redistribution of cholesterol to an esterified cholesterol pool [30]. While our current work demonstrated that in the absence of Ch25h, there was increased binding and uptake of *S. pneumoniae*, it will be important for future studies to determine the role of cholesterol redistribution on the sensitivity of macrophages to *S. pneumoniae* infection.

In the absence of Ch25h, cholesterol dysregulation and defective efferocytosis contributed to increased LPS-mediated lung injury [13]. Importantly, *Ch25h* expression was induced in efferocytic macrophages and was required for *Mertk* upregulation [13]. Interestingly, in response to *S. pneumoniae* infection, we observed a role for Ch25h in mediating phagocytic receptor expression, with no significant impact on *Mertk* expression. In contrast to LPS or *Klebsiella pneumoniae*, it is possible that during *S. pneumoniae* infection, Ch25h and 25-HC, through changes in cholesterol metabolism may play an important role in mediating the macrophage “switch” between phagocytic and efferocytic signaling. While our current findings demonstrate increased phagocytic processing and clearance of *S. pneumoniae* occur in the absence of Ch25h, future work will need to be performed to investigate mechanisms that underlie macrophage efferocytic/phagocytic signaling.

Taken together, our findings demonstrate the important role of Ch25h in modulating host responsiveness to *S. pneumoniae*.

## Data Availability

Data presented in this study are provided within the article.

## Supplementary Materials

The following supporting information can be downloaded at: https://www.mdpi.com/article/10.3390/cells12040570/s1, Figure S1: Gating strategy for alveolar macrophage phagocytosis of Alexa Fluor 647^+^ FITC labeled *S. pneumoniae*.; Figure S2: TEM images of wild-type and *Ch25h^−/−^* alveolar macrophages treated with media.

## References

[1] Ortqvist A., Hedlund J., Kalin M. (2005). *Streptococcus pneumoniae*: Epidemiology, risk factors, and clinical features. Semin. Respir. Crit. Care Med..

[2] Gotts J.E., Bernard O., Chun L., Croze R.H., Ross J.T., Nesseler N., Wu X., Abbott J., Fang X., Calfee C.S. (2019). Clinically relevant model of pneumococcal pneumonia, ARDS, and nonpulmonary organ dysfunction in mice. Am. J. Physiol. Cell. Mol. Physiol..

[3] Aberdein J.D., Cole J., Bewley M., Marriott H.M., Dockrell D.H. (2013). Alveolar macrophages in pulmonary host defence the unrecognized role of apoptosis as a mechanism of intracellular bacterial killing. Clin. Exp. Immunol..

[4] Knapp S., Leemans J.C., Florquin S., Branger J., Maris N.A., Pater J., van Rooijen N., van der Poll T. (2003). Alveolar macrophages have a protective antiinflammatory role during murine pneumococcal pneumonia. Am. J. Respir. Crit. Care Med..

[5] Maxfield F.R., Tabas I. (2005). Role of cholesterol and lipid organization in disease. Nature.

[6] Yan J., Horng T. (2020). Lipid Metabolism in Regulation of Macrophage Functions. Trends Cell Biol..

[7] Walther T.C., Farese V.R. (2012). Lipid droplets and cellular lipid metabolism. Annu. Rev. Biochem..

[8] Afonso M.S., Machado R.M., Lavrador M.S., Quintao E.C.R., Moore K.J., Lottenberg A.M. (2018). Molecular Pathways Underlying Cholesterol Homeostasis. Nutrients.

[9] Goldstein J.L., Rawson B.R., Brown S.M. (2002). Mutant mammalian cells as tools to delineate the sterol regulatory element-binding protein pathway for feedback regulation of lipid synthesis. Arch. Biochem. Biophys..

[10] Bauman D.R., Bitmansour A.D., McDonald J.G., Thompson B.M., Liang G., Russell D.W. (2009). 25-Hydroxycholesterol secreted by macrophages in response to Toll-like receptor activation suppresses immunoglobulin A production. Proc. Natl. Acad. Sci. USA.

[11] Diczfalusy U., Olofsson K.E., Carlsson A.-M., Gong M., Golenbock D.T., Rooyackers O., Fläring U., Björkbacka H. (2009). Marked upregulation of cholesterol 25-hydroxylase expression by lipopolysaccharide. J. Lipid Res..

[12] Karuna R., Christen I., Sailer A.W., Bitsch F., Zhang J. (2015). Detection of dihydroxycholesterols in human plasma using HPLC-ESI-MS/MS. Steroids.

[13] Madenspacher J.H., Morrell E.D., Gowdy K.M., McDonald J.G., Thompson B.M., Muse G.W., Martinez J., Thomas S.Y., Mikacenic C., Nick J.A. (2020). Cholesterol 25-hydroxylase promotes efferocytosis and resolution of lung inflammation. JCI Insight.

[14] Blanc M., Hsieh W.Y., Robertson K.A., Kropp K.A., Forster T., Shui G., Lacaze P., Watterson S., Griffiths S.J., Spann N.J. (2013). The transcription factor STAT-1 couples macrophage synthesis of 25-hydroxycholesterol to the interferon antiviral response. Immunity.

[15] Park K., Scott L.A. (2010). Cholesterol 25-hydroxylase production by dendritic cells and macrophages is regulated by type I interferons. J. Leukoc. Biol..

[16] Zhang X., Yang W., Wang X., Zhang W., Tian H., Deng H., Zhang L., Gao G. (2018). Identification of new type I interferon-stimulated genes and investigation of their involvement in IFN-beta activation. Protein Cell.

[17] Liu Y., Hulten L.M., Wiklund O. (1997). Macrophages isolated from human atherosclerotic plaques produce IL-8, and oxysterols may have a regulatory function for IL-8 production. Arter. Thromb. Vasc. Biol..

[18] Lemaire-Ewing S., Berthier A., Royer M.C., Logette E., Corcos L., Bouchot A., Monier S., Prunet C., Raveneau M., Rébé C. (2009). 7β-Hydroxycholesterol and 25-hydroxycholesterol-induced interleukin-8 secretion involves a calcium-dependent activation of c-fos via the ERK1/2 signaling pathway in THP-1 cells: Oxysterols-induced IL-8 secretion is calcium-dependent. Cell Biol. Toxicol..

[19] Fu H., Spieler F., Großmann J., Riemann D., Larisch M., Hiebl B., Schlecht K., Jaschke C., Bartling B., Hofmann B. (2014). Interleukin-1 potently contributes to 25-hydroxycholesterol-induced synergistic cytokine production in smooth muscle cell-monocyte interactions. Atherosclerosis.

[20] Pokharel S.M., Shil N.K., Gc J.B., Colburn Z.T., Tsai S.-Y., Segovia J.A., Chang T.-H., Bandyopadhyay S., Natesan S., Jones J.C.R. (2019). Integrin activation by the lipid molecule 25-hydroxycholesterol induces a proinflammatory response. Nat. Commun..

[21] Gold E.S., Diercks A.H., Podolsky I., Podyminogin R.L., Askovich P.S., Treuting P.M., Aderem A. (2014). 25-Hydroxycholesterol acts as an amplifier of inflammatory signaling. Proc. Natl. Acad. Sci. USA.

[22] Jang J., Park S., Hur H.J., Cho H.-J., Hwang I., Kang Y.P., Im I., Lee H., Lee E., Yang W. (2016). 25-hydroxycholesterol contributes to cerebral inflammation of X-linked adrenoleukodystrophy through activation of the NLRP3 inflammasome. Nat. Commun..

[23] Koarai A., Yanagisawa S., Sugiura H., Ichikawa T., Kikuchi T., Furukawa K., Akamatsu K., Hirano T., Nakanishi M., Matsunaga K. (2012). 25-Hydroxycholesterol enhances cytokine release and Toll-like receptor 3 response in airway epithelial cells. Respir. Res..

[24] Sugiura H., Koarai A., Ichikawa T., Minakata Y., Matsunaga K., Hirano T., Akamatsu K., Yanagisawa S., Furusawa M., Uno Y. (2012). Increased 25-hydroxycholesterol concentrations in the lungs of patients with chronic obstructive pulmonary disease. Respirology.

[25] Liu C., Yang X.V., Wu J., Kuei C., Mani N.S., Zhang L., Yu J., Sutton S.W., Qin N., Banie H. (2011). Oxysterols direct B-cell migration through EBI2. Nature.

[26] Zou T., Garifulin O., Berland R., Boyartchuk V.L. (2011). Listeria monocytogenes infection induces prosurvival metabolic signaling in macrophages. Infect. Immun..

[27] Zang R., Case J.B., Yutuc E., Ma X., Shen S., Castro M.F.G., Liu Z., Zeng Q., Zhao H., Son J. (2020). 25-hydroxylase suppresses SARS-CoV-2 replication by blocking membrane fusion. Proc. Natl. Acad. Sci. USA.

[28] Wang S., Li W., Hui H., Tiwari S.K., Zhang Q., Croaler B.A., Rawlings S., Smith D., Carlin A.F., Rana T.M. (2020). Cholesterol 25-Hydroxylase inhibits SARS-CoV-2 and other coronaviruses by depleting membrane cholesterol. EMBO J..

[29] Yuan Y., Wang Z., Tian B., Zhou M., Fu Z.F., Zhao L. (2019). Cholesterol 25-hydroxylase suppresses rabies virus infection by inhibiting viral entry. Arch. Virol..

[30] Zhou Q.D., Chi X., Lee M.S., Hsieh W.Y., Mkrtchyan J., Feng A.-C., He C., York A.G., Bui V.L., Kronenberger E.B. (2020). Interferon-mediated reprogramming of membrane cholesterol to evade bacterial toxins. Nat. Immunol..

[31] Bottemanne P., Paquot A., Ameraoui H., Guillemot-Legris O., Alhouayek M., Muccioli G.G. (2021). 25-Hydroxycholesterol metabolism is altered by lung inflammation, and its local administration modulates lung inflammation in mice. FASEB J..

[32] Misharin A.V., Morales-Nebreda L., Mutlu G.M., Budinger G.R.S., Perlman H. (2013). Flow cytometric analysis of macrophages and dendritic cell subsets in the mouse lung. Am. J. Respir. Cell Mol. Biol..

[33] Sun F., Xiao G., Qu Z. (2017). Murine Bronchoalveolar Lavage. Bio. Protoc..

[34] Halbower A.C., Mason R.J., Abman S.H., Tuder R.M. (1994). Agarose infiltration improves morphology of cryostat sections of lung. Lab. Investig..

[35] Reboldi A., Dang E.V., Mcdonald J.G., Liang G., Russell D.W., Cyster J.G. (2014). Inflammation. 25-Hydroxycholesterol suppresses interleukin-1-driven inflammation downstream of type I interferon. Science.

[36] Domon H., Oda M., Maekawa T., Nagai K., Takeda W., Terao Y. (2016). *Streptococcus pneumoniae* disrupts pulmonary immune defence via elastase release following pneumolysin-dependent neutrophil lysis. Sci. Rep..

[37] Abrams M.E., Johnson K.A., Perelman S.S., Zhang L.-S., Endapally S., Mar K.B., Thompson B.M., McDonald J.G., Schoggins J.W., Radhakrishnan A. (2020). Oxysterols provide innate immunity to bacterial infection by mobilizing cell surface accessible cholesterol. Nat. Microbiol..

[38] van Lieshout M.H.P., de Vos A.F., Dessing M.C., de Porto A.P.N.A., de Boer O.J., de Beer R., Terpstra S., Florquin S., van’t Veer C., van der Poll T. (2018). ASC and NLRP3 impair host defense during lethal pneumonia caused by serotype 3 *Streptococcus pneumoniae* in mice. Eur. J. Immunol..

[39] Phipps J.C., Aronoff D.M., Curtis J.L., Goel D., O’Brien E., Mancuso P. (2010). Cigarette smoke exposure impairs pulmonary bacterial clearance and alveolar macrophage complement-mediated phagocytosis of *Streptococcus pneumoniae*. Infect. Immun..

[40] Cruz C.S.D., Liu W., He C.H., Jacoby A., Gornitzky A., Ma B., Flavell R., Lee C.G., Elias J.A. (2012). Chitinase 3-like-1 promotes *Streptococcus pneumoniae* killing and augments host tolerance to lung antibacterial responses. Cell Host Microbe.

[41] Paudel S., Baral P., Ghimire L., Bergeron S., Jin L., Decorte J.A., Le J.T., Cai S., Jeyaseelan S. (2019). CXCL1 regulates neutrophil homeostasis in pneumonia-derived sepsis caused by *Streptococcus pneumoniae* serotype 3. Blood.

[42] Spreer A., Kerstan H., Böttcher T., Gerber J., Siemer A., Zysk G., Mitchell T.J., Eiffert H., Nau R. (2003). Reduced release of pneumolysin by *Streptococcus pneumoniae* in vitro and in vivo after treatment with nonbacteriolytic antibiotics in comparison to ceftriaxone. Antimicrob. Agents Chemother..

[43] Hostetter M.K. (1986). Serotypic variations among virulent pneumococci in deposition and degradation of covalently bound C3b: Implications for phagocytosis and antibody production. J. Infect. Dis..

[44] Neeleman C., Geelen S.P., Aerts P.C., Daha M.R., Mollnes T.E., Roord J.J., Posthuma G., Van Dijk H., Fleer A. (1999). Resistance to both complement activation and phagocytosis in type 3 pneumococci is mediated by the binding of complement regulatory protein factor H. Infect. Immun..

