# Supplementary material for: Role of Cholesterol 25-Hydroxylase (Ch25h) in Mediating Innate Immune Responses to Streptococcus pneumoniae Infection
Source: Cells. 2023 Feb 10;12(4):570. doi: 10.3390/cells12040570 (PMC9953875; doi:10.3390/cells12040570)
Supplement: Supplementary file 1 [file cells-12-00570-s001.zip › cells-2144922-supplementary.pdf]

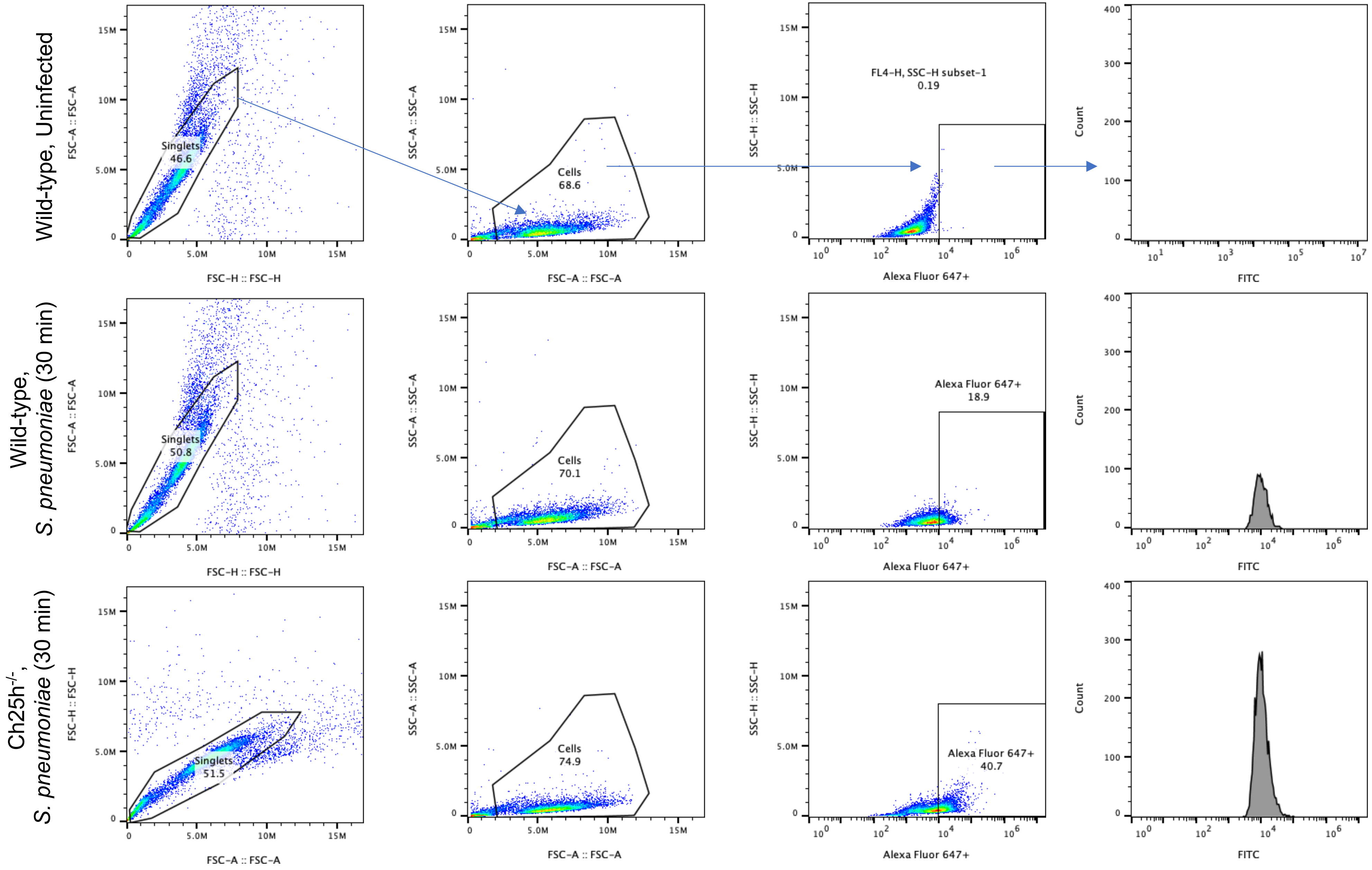

Supplemental Figure S1: Gating strategy for alveolar macrophage phagocytosis of Alexa Fluor 647⁺ FITC labeled *S. pneumoniae*. Representative control and 30-minute samples are shown.

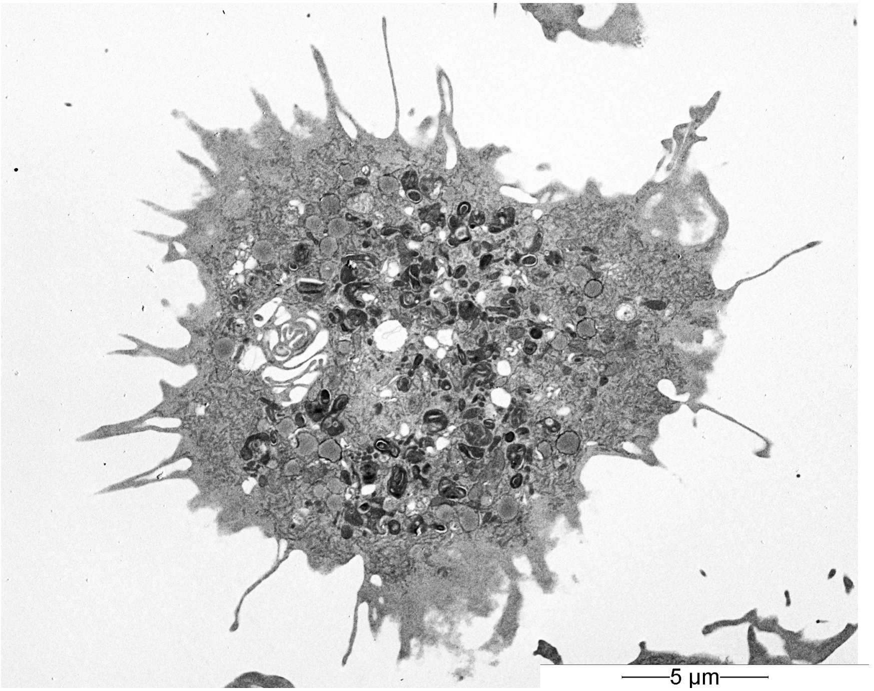

Wild-type, Media Treated

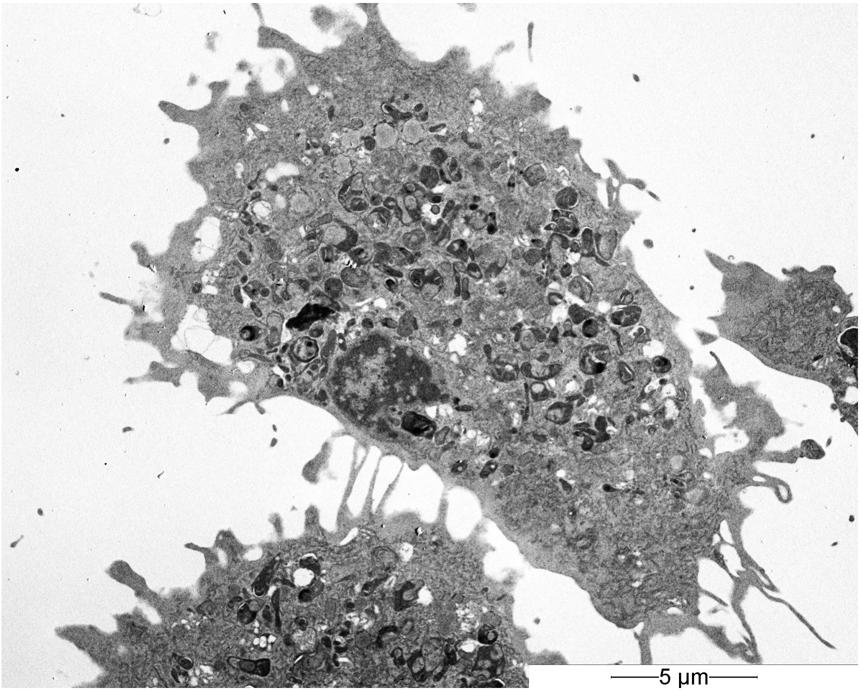

Ch25h$^{-/-}$, Media Treated

Supplemental Figure S2: Wild-type and *Ch25h*$^{-/-}$ alveolar macrophages treated with media.
